# Consumers’ decisions to access or avoid added sugars information on the updated Nutrition Facts label

**DOI:** 10.1371/journal.pone.0249355

**Published:** 2021-03-29

**Authors:** Elizabeth Jiyoon Kim, Brenna Ellison, Brandon McFadden, Melissa Pflugh Prescott

**Affiliations:** 1 Department of Agricultural and Consumer Economics, University of Illinois at Urbana-Champaign, Urbana, Illinois, United States of America; 2 Department of Applied Economics and Statistics, University of Delaware, Newark, Delaware, United States of America; 3 Department of Food Science and Human Nutrition, University of Illinois at Urbana-Champaign, Urbana, Illinois, United States of America; University of Rhode Island, UNITED STATES

## Abstract

The Nutrition Facts (NF) label was recently updated and now includes the added sugars content in an effort to reduce added sugars consumption. This study investigated whether consumers wanted to access or avoid the added sugars content using an online experiment and five product categories (yogurt, cereal, fruit juice, snack bar, ice cream). We recruited a sample of 490 U.S. adults (49% female; 73% White/Caucasian). Respondents were randomly assigned to an information treatment (simple or full) before making decisions on whether to access or avoid the added sugars content. The simple information treatment explained that added sugars information was now available on the NF label, while the full information treatment included additional details (e.g., how to interpret the added sugars content and associated diseases). After making the access or avoid decisions for each product category, respondents rated their likelihood of purchase for ten products (two per category). Rates of information avoidance were much lower than what has been observed in previous studies, and rates of avoidance did not vary by information treatment. The majority of respondents (75–87% across the five product categories) preferred to access the added sugars content. Still, we found some consumers preferred to avoid this information, with higher rates of avoidance for the ice cream product category. Additionally, we found significant differences in likelihood of purchase ratings between information accessors and avoiders. Respondents who chose to access the added sugars information exhibited healthier purchasing behaviors for all product categories; they were more likely to purchase low added sugars products and less likely to purchase high added sugars products relative to information avoiders. Given consumers’ demonstrated interest in accessing the added sugars content, it is important that the new changes to the NF label be broadly communicated to promote healthy eating behaviors.

## Introduction

Obesity is a prevalent health problem in the U.S.; almost 75% of US adults are classified as overweight or obese [1, 2]. Added sugars, which are found in products like sweetened beverages, bakery products, and ice cream, have been identified as a key contributor to obesity in the U.S. [3]. They are defined as sugars added to foods during processing, preparation, and at the table, like sucrose, brown sugar, high fructose corn syrup, or honey [4]. The 2020–2025 Dietary Guidelines for Americans (DGA) recommends that added sugars comprise no more than 10% of total daily energy intake. Yet, on average, Americans currently exceed this recommendation [5]. The scientific literature has consistently found a causal link between excessive consumption of added sugars and obesity as well as diabetes and cardiovascular disease [6, 7]. As a result, several studies have called for the inclusion of added sugars content in the Nutrition Facts (NF) label.

In 2016, the U.S. Food and Drug Administration (FDA) released an updated NF label, which required food manufacturers to provide the added sugars content, among other changes (large firms with sales of $10 million or more had to comply by January 1, 2020, and small firms have until January 1, 2021). In the updated NF label, the added sugars content is now provided as a sub-component below total sugars; it is displayed in grams with the accompanying Percent Daily Value [8]. For more information on how this format was selected by FDA, see the proposed and final rules on the provision of added sugars in the updated NF label [8, 9].

Before updating the NF label, few studies explored consumers’ perception of added sugars. Multiple studies found that consumers struggled to accurately interpret the added sugars information [10–12]. In a more recent study, Khandpur, Rimm, and Moran found that consumers’ comprehension of added sugars content was improved under the updated NF label and that generally consumers supported disclosure of the added sugars information [13].

Providing consumers with additional nutrition information has the potential to improve their food selection and/or consumption behaviors. Previous studies have found that NF label use is associated with food choices that are lower in cholesterol [14], sugar, total fat, saturated fat [15], and added sugar [16]. Other research suggests some consumers may actively avoid nutrition information [17, 18].

For added sugars, it is unclear whether consumers will seek or avoid this information. There are a few potential explanations for why consumers may avoid this information. First, consumers may want to avoid any guilt or regret associated with consuming foods with added sugars that may be viewed as unhealthy. Second, consumers may want to avoid added sugars information for products they believe to be healthy in the event that acquiring the information would be inconsistent with their beliefs [18]. If consumers willfully ignore the added sugars information, the intended effects (e.g., improved food choice and, ultimately, health outcomes) of including this information may not be realized.

The primary goal of this study was to investigate whether consumers wanted to access the newly included added sugars information in the updated NF label when purchasing food products. While recent research indicates that including added sugars information does not affect food choice [19], choosing to acquire that information is a necessary condition for changing behavior. In this study, we explored two factors that may influence information acquisition. The first factor relates to consumers’ understanding of added sugars and why this information is important. Some consumers may struggle to correctly identify what added sugars are and what products they are in [13, 20, 21] and may not know which diseases are associated with the overconsumption of added sugars [22]. These knowledge gaps may decrease the likelihood that consumers choose to acquire the added sugars information. In this study, we randomly assigned respondents to either a simple or full information treatment that varied the amount of information provided on added sugars. We hypothesized that respondents who received the full information treatment (which addresses the knowledge gaps identified in the literature) would be more likely to acquire the added sugars information. The second factor that may influence information acquisition is product type. Grebitus and Davis found that attention to the NF label is affected by the healthfulness of a food product category, possibly because nutrition information is a source of disutility when selecting and consuming some foods [23]. Similarly, we hypothesized that consumers were more likely to avoid the added sugars information for high-sugar products.

## Methods

### Sample recruitment

To test consumers’ willful avoidance of added sugars information on the updated NF label, an online experiment was conducted using the Qualtrics survey platform in April, 2020. The study was approved by the University of Illinois at Urbana-Champaign Institutional Review Board (IRB #20576). We recruited a sample of 490 U.S. residents who were over the age of 18. Given our group sizes (*n* = 243 for simple and *n* = 247 for full information treatments, respectively), we had sufficient power (0.80) to detect a 12-percentage point difference in the rate of information avoidance between the two treatments at a 95% confidence level. Our sample was recruited using Prolific, which is an online crowdsourcing platform that provides a higher transparency about the subject pool for research than other online platforms [24]. Prolific outperforms other online panels in terms of data quality [25]. On average, Prolific recruits more diverse, more naïve, and less dishonest participants relative to Amazon MTurk [25]. Respondents were compensated $1.30 for completion of the study.

### Experimental design

**Fig 1:**
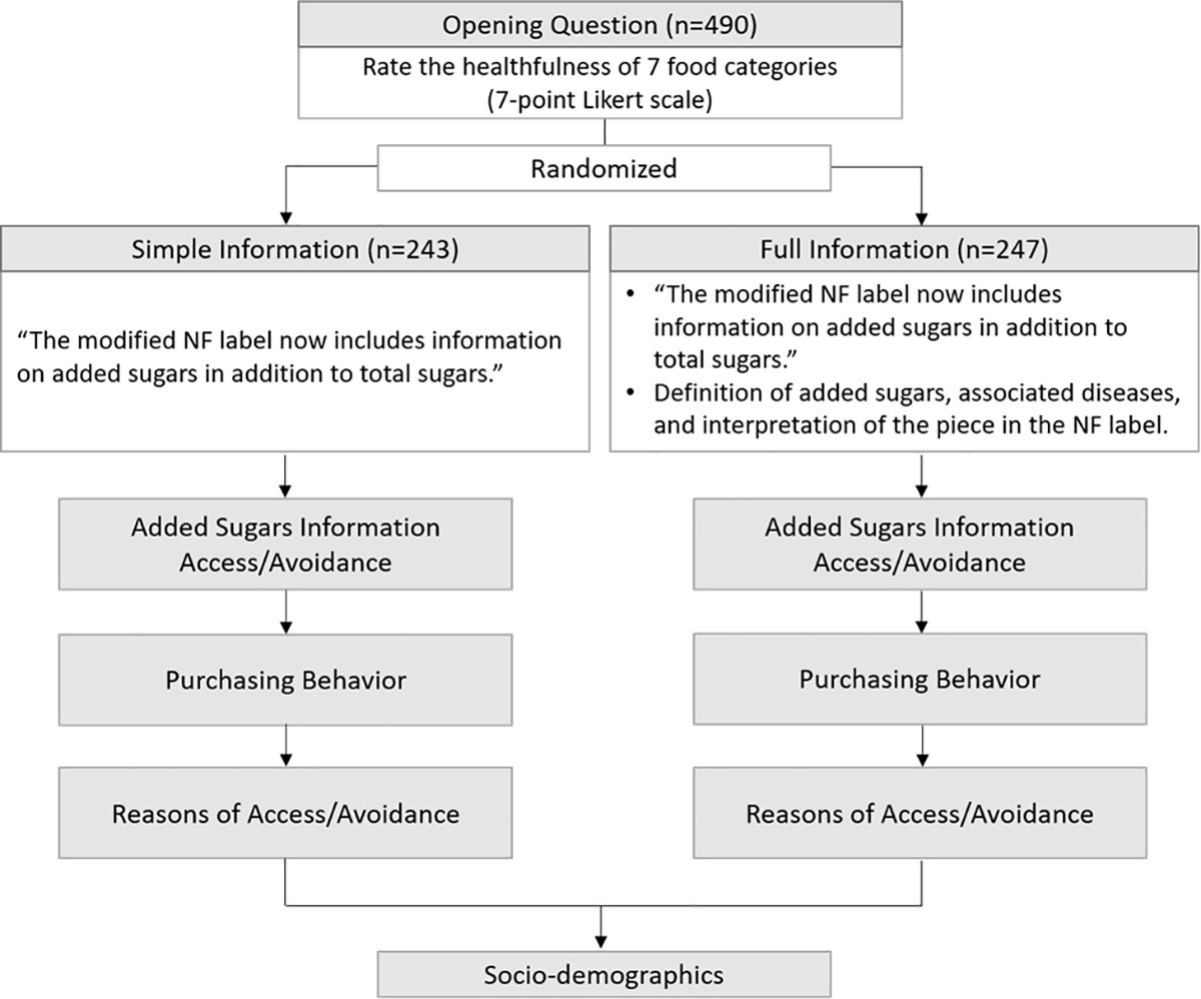
Experimental design.

**Fig 2:**
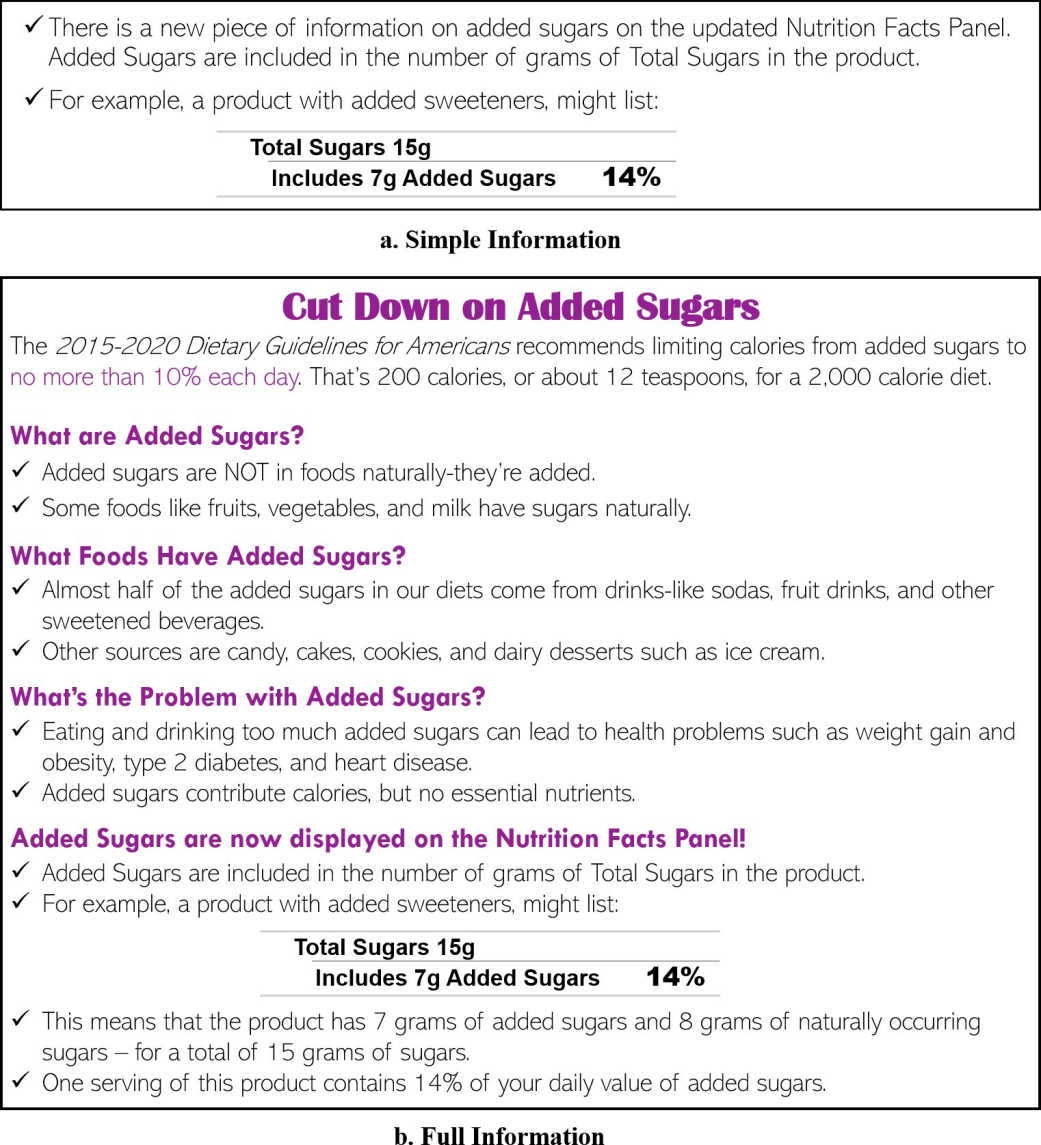
Simple (panel a) and full (panel b) information treatments.

After providing consent, respondents were first asked to rate the healthfulness of seven food categories, including yogurt, fruit juice, fresh fruit, ice cream, snack bar, soda, and cereal on a 7-point scale (1 = very unhealthy to 7 = very healthy) as an opening question to ensure respondents perceived differences in the healthfulness of products. Fresh fruit and soda were included to assess how respondents rated their healthfulness in relation to the target products used in this study. The researchers selected these products as polar examples of the healthfulness scale, and respondents rated them as expected. Respondents were thereafter randomly assigned into a simple or full information treatment (see Fig 1). Fig 2 displays the added sugars information provided for each treatment. The simple information treatment informed respondents that the added sugars content was now included in the NF label and showed an exemplar of the NF label. Respondents in the full information treatment were provided with more information including the definition of added sugars, the recommended daily amount of added sugars intake, example foods that contain added sugars, diseases associated with overconsumption of added sugars, and how to interpret the added sugars information in the NF label.

Regardless of information treatment, all respondents were then asked whether or not they would like to receive the added sugars content for five product categories to assess individuals’ avoidance of added sugars information. We chose yogurt, cereal, fruit juice, snack bar, and ice cream as our target product categories. We selected products that were identified as primary sources of added sugars in the 2015–2020 DGA (note: in the fruit juice category, 100% fruit juice does not contain added sugars, but fruit juice cocktails do; we included both in the present study). To be clear, information acquisition or avoidance decisions were made by respondents for each product category to allow for heterogeneity in acquisition and avoidance behavior.

After making their information acquisition or avoidance decisions, respondents were asked to rate the likelihood of purchase for 10 products (two products per category; one product with a low level of added sugars and another with a high level of added sugars) on a 7-point scale (1 = Not at all likely to 7 = Extremely likely). Low and high levels of added sugars varied by product category. The researchers did not set objective thresholds (e.g., a product with no more than X grams of added sugars is considered low), primarily because it was difficult to find products that met such thresholds across all five categories. Rather, the researchers tried to select products that contained a low or high level of added sugars, by proportion, to the total sugars content. See S1 Table for a list of the 10 products and their added sugars content. Product order was randomized across respondents, and respondents were shown each product individually. The high- and low-sugar versions of a product within the same category were not shown side by side nor were they evaluated consecutively (unless randomized that way by chance). Respondents who chose to see the added sugars information received the added and total sugars information (in grams) as displayed in the updated NF label in addition to an image of the product. Respondents who chose to avoid the information only saw the product image. Product images were only shown when respondents were asked to rate their likelihood of purchase for each product.

Subjects were then asked to provide the main reason for wanting or not wanting the added sugars information for each product category. We adapted the responses from Thunström [26] who investigated the avoidance of calorie information in a restaurant setting. Similar to the Thunström paper [26], there were more potential reasons for information avoidance compared to information acquisition (eight and five, respectively). Example reasons for information avoidance included ‘I don’t want to think about added sugars when purchasing this product’; ‘I would enjoy this product less if I knew the added sugars content’; and ‘I would not want to know the added sugars content because it would not matter to my food choice anyway’. Example reasons for information acquisition included ‘The added sugars content would matter to my food choice’ and ‘The added sugars information would not affect my food choice, but I would be curious to know’. At the end of the survey, respondents completed a label use behavior question and socio-demographic questions.

### Data analysis

To estimate determinants of consumers’ willful avoidance of added sugars information, we employed the following model:

$$Avoid_{ij}=f(Full_{i},X_{i})$$ (1)

where *avoid_ij_* was coded as one if subject *i* chose to avoid the added sugars information for product category *j* (1 = yogurt, 2 = cereal, 3 = fruit juice, 4 = snack bar, and 5 = ice cream). *Full_i_* was an indicator variable equal to one if subjects were randomized into the full information treatment. A negative *Full_i_* coefficient could be interpreted as an educational effect on consumers’ willful avoidance of added sugars information. This means that consumers provided with the full information treatment were less willing to avoid the added sugars information compared to those who received the simple information treatment. A vector of demographic variables, *X_i_*, included primary shopper, frequency of grocery shopping, sex, age, household income, household size, living with a child(ren) under 18 years old, race, whether on a special diet, family history of diet-related diseases, BMI classification, and label use behavior.

The study estimated determinants of avoidance of added sugars information using a multivariate probit model. The multivariate probit allowed for simultaneous estimation of Eq (1) for all five product categories and included the estimation of pairwise correlations across the errors of the five equations [27]. The multivariate probit model estimated a set of probabilities depending on whether the subject *i* wanted to access or avoid the added sugars for one product category and their desire to access or avoid it for the other categories. We tested the assumption that the error terms across equations were uncorrelated (null hypothesis in the multivariate probit model) using a Likelihood Ratio test.

Another interest of this study was to test how consumers’ avoidance of added sugars information related to their likelihood of purchase. One-way analysis of variance (ANOVA) was used to examine mean differences in the likelihood of purchase for each product, comparing information accessors and information avoiders. All analyses were conducted using the statistical software package STATA version 16.0.

## Results

Table 1 summarizes the sample characteristics. The majority of respondents (84%) served as the primary shopper in their household. Our sample was comparable to the U.S. population in terms of sex; however, our respondents were younger and more educated relative to the U.S. population [28].

**Table 1:** Summary of sample characteristics.

Variable	Sample Proportion (%) or Mean±SD	
	Simple information treatment (n = 243)	Full information treatment (n = 247)
Primary shopper in the household	84.30%	83.80%
Sex		
Male	55.60%	47.00%
Female	44.40%	53.00%
Age		
18–34 years	30.00%	32.40%
35–54 years	35.40%	31.20%
55 years or older	34.60%	36.40%
Household income		
Less than $50,000	42.80%	44.10%
$50,000 - $99,999	37.40%	34.80%
$100,000 or more	19.80%	21.10%
Education: Bachelor’s degree or higher	55.60%	63.20%
Race		
White/Caucasian	75.70%	70.90%
Black/African American	10.70%	13.40%
Asian/Pacific Islander	6.60%	5.70%
Hispanic or Latino/a	4.90%	6.90%
Other	2.10%	3.10%
Number of household members	2.57±1.32	2.52±1.30
Child under 18 present in household	24.30%	23.50%
Participation in any food assistance program (SNAP, WIC, etc.)	9.90%	10.50%
Completed a course in health or nutrition	30.50%	34.40%
On a special diet monitoring intake of a specific nutrient(s)	12.30%	18.60%
Family history of an added-sugars related disease (diabetes, obesity, heart disease)	69.50%	60.70%
Obese: Reported BMI score greater than 30	28.90%	28.30%
Reads Nutrition Facts label most of the time or always when buying a food product for the first time	69.10%	72.50%

Note: There were no differences in participant characteristics across information treatments with the exception of the family history of an added-sugars related disease variable (*p* = 0.041).

### Information avoidance behavior

**Fig 3:**
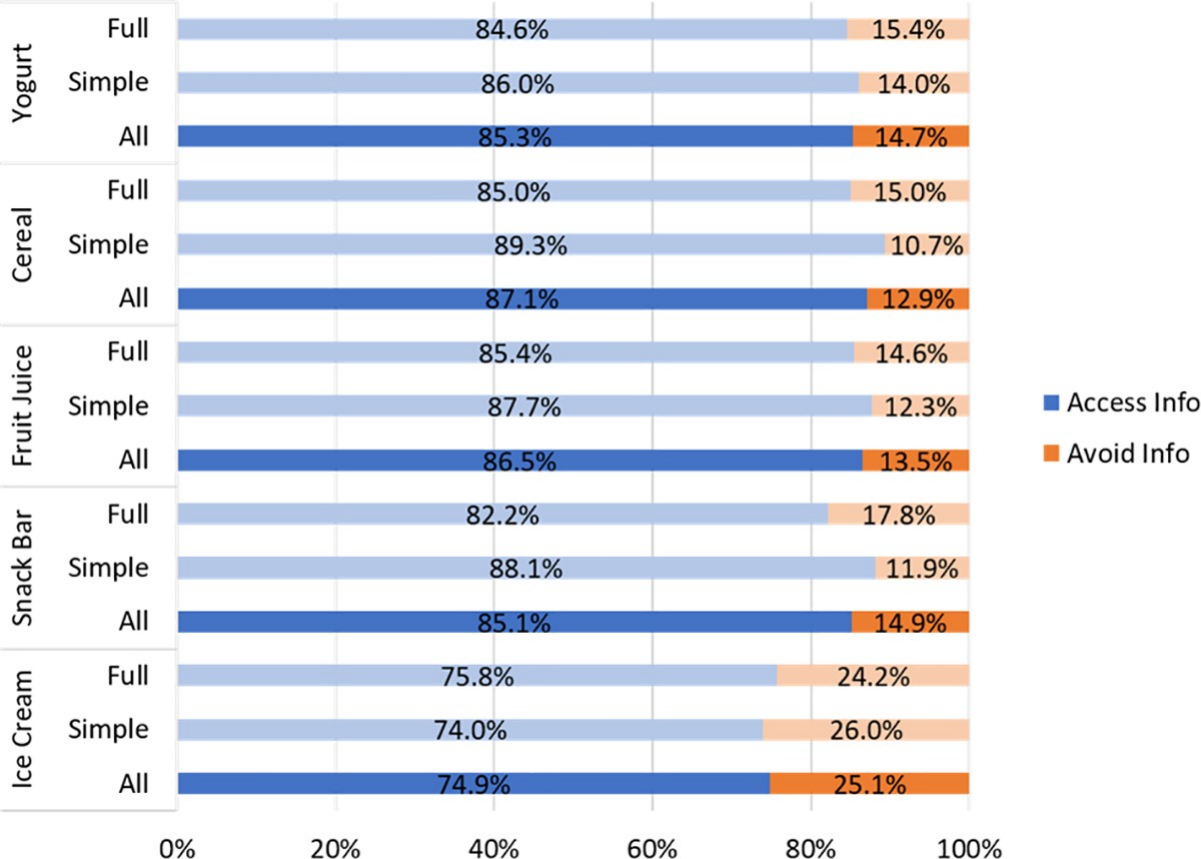
Percentage of added sugars information accessors and avoiders by product category and information treatment (N = 490).

First, we examined consumers’ decisions to access or avoid the added sugars information. Fig 3 presents the shares of added sugars information accessors and avoiders for each product category by information treatment and for all participants combined. While we hypothesized that consumers in the full information would be more likely to access the added sugars information, there were no significant differences in the rates of access/avoidance across the two information treatments (Fig 3). However, consumers’ information avoidance behavior varied by product category as expected. In particular, the rate of avoidance of added sugars information for ice cream (25.1%) was significantly higher (all p-values from t-tests < 0.001) than that of yogurt (14.7%), cereal (12.9%), fruit juice (13.5%), and snack bar (14.9%). There were no significant differences in avoidance across non-ice cream categories.

Respondents reported their primary reasons for wanting to access or avoid the added sugars content for each product category. See S2 and S3 Tables for access and avoidance results, respectively. For all categories, the majority of respondents who wanted the information (47–66% across the five product categories) stated that the added sugars content would matter to their food choices. A smaller share of respondents (22–38% across the five product categories) stated that they would be interested to know the added sugars content but indicated it would not affect their food choice. Less than 10% of respondents indicated that they would enjoy the product more if they knew the added sugars content.

There was some heterogeneity in the reasons for avoiding information across product categories. For yogurt, snack bar, and ice cream, a little less than one-third (29%) of respondents reported avoiding the information because it would not matter to their food choice anyway. For ice cream, an additional 23% (12–19% for other product categories) stated that they didn’t want to think about added sugars when purchasing this product. Across all products, approximately 10–20% of respondents stated they chose to avoid the information because a) it would make them feel guilty or b) they would enjoy the product less if they knew the added sugars information.

Table 2 reports the results of the multivariate probit estimation. The correlation coefficients at the bottom of Table 2 were all positive and significant. The null hypothesis of uncorrelated error terms across five equations was rejected, meaning the multivariate probit model was preferred to separate estimation of individual probit models for each product category. The positive coefficients implied the potential complementarities across the five product categories. Consumers who avoided added sugars information for yogurt, for example, were shown to also be more likely to avoid it when purchasing cereal, fruit juice, snack bar, or ice cream.

**Table 2:** Predictors of information avoidance behavior by product category (N = 484).

Variable	(1) Yogurt	(2) Cereal	(3) Fruit Juice	(4) Snack Bar	(5) Ice Cream
Full Information Treatment	0.119 (0.148)	0.180 (0.150)	0.139 (0.149)	0.334** (0.145)	0.168 (0.128)
Primary Shopper	0.076 (0.219)	-0.098 (0.207)	-0.075 (0.213)	0.057 (0.204)	-0.396** (0.173)
Sex: Female	0.035 (0.149)	0.102 (0.151)	-0.052 (0.150)	0.073 (0.144)	-0.025 (0.129)
Age: 35–54 years	0.167 (0.200)	-0.274 (0.200)	0.254 (0.207)	0.184 (0.194)	0.194 (0.169)
Age: 55 years or older	0.321* (0.193)	0.023 (0.189)	0.546*** (0.2020)	0.221 (0.189)	0.054 (0.170)
Bachelor’s degree or higher	-0.315** (0.159)	-0.130 (0.160)	-0.126 (0.163)	-0.156 (0.159)	-0.044 (0.141)
Income: $50,0000 - $99,999	0.281 (0.181)	-0.046 (0.185)	-0.007 (0.191)	0.245 (0.183)	0.079 (0.161)
Income: $100,000 or more	-0.129 (0.238)	-0.203 (0.227)	0.168 (0.217)	0.145 (0.210)	0.047 (0.188)
Number in Household	-0.061 (0.083)	-0.043 (0.082)	-0.046 (0.081)	0.072 (0.074)	-0.042 (0.068)
Children in Household	0.084 (0.243)	0.357 (0.234)	0.313 (0.232)	-0.152 (0.228)	0.278 (0.203)
Race: White/Caucasian	-0.221 (0.161)	-0.125 (0.163)	-0.106 (0.164)	-0.159 (0.157)	0.197 (0.146)
Nutrition Assistance Program	0.238 (0.235)	0.161 (0.241)	0.430* (0.228)	0.246 (0.233)	0.431** (0.216)
Special Diet	-0.075 (0.215)	-0.232 (0.235)	-0.413* (0.231)	-0.379* (0.224)	-0.120 (0.184)
Family History	-0.005 (0.160)	-0.079 (0.160)	-0.072 (0.157)	-0.055 (0.153)	0.297** (0.142)
Obese (BMI greater than 30)	0.128 (0.162)	0.093 (0.167)	-0.094 (0.169)	0.085 (0.163)	-0.051 (0.145)
Frequently Reads NF Label	-0.473*** (0.158)	-0.255 (0.159)	0.018 (0.163)	-0.383** (0.154)	-0.173 (0.140)
Constant	-0.729* (0.382)	-0.662* (0.372)	-1.166*** (0.378)	-1.216*** (0.364)	-0.708** (0.320)
*Correlation Coefficients*					
Yogurt		0.726***	0.701***	0.639***	0.576***
Cereal			0.704***	0.796***	0.651***
Fruit Juice				0.688***	0.635***
Snack Bar					0.735***

Notes: Dependent variables were coded as one for each product category if respondents chose to avoid the added sugars content. Six responses were excluded from analyses for incomplete demographic information. Age categories relative to those 18–34 years old. Income categories relative to those with income less than $50,000. Race category relative to all other races. Log-likelihood of the multivariate probit estimation was -800.885. Standard errors in parentheses. Significance is denoted by *, **, *** for 10%, 5%, and 1% levels, respectively.

Socio-demographics had little explanatory power for information avoidance decisions. While there were few consistently significant findings across the majority of products, we found that respondents who frequently use the NF label when purchasing new products were less likely to avoid the added sugars information (significant for yogurt and snack bar). Additionally, older respondents were shown to be more likely to avoid the added sugars information (significant for yogurt and fruit juice).

We found that those who have participated in any nutrition assistance program were more likely to avoid the added sugars content (significant for fruit juice and ice cream). In addition, individuals with a family history of added sugars-related diseases were less likely to avoid the added sugars information, except for the ice cream product category, which had a positive and significant coefficient.

### Likelihood of purchase

Table 3 compares the likelihood of purchase ratings for the 10 products between information accessors and information avoiders. For all product categories, information accessors exhibited higher likelihood of purchase ratings, on average, for low-added sugars products compared to information avoiders (all significantly different except low-added sugar yogurt and snack bar). Further, we observed the opposite for high-added sugars products. Information accessors reported lower likelihood of purchase ratings, on average, than information avoiders (all significantly different).

**Table 3:** One-Way ANOVA tests comparing likelihood of purchase ratings between information accessors and information avoiders by product.

Product	Mean Likelihood of Purchase ± SD		P-value
	Information Accessors	Information Avoiders	
Yogurt	n = 418	n = 72	
Low-AS Product	4.08 ± 1.85	4.06 ± 2.34	0.909
High-AS Product	3.75 ± 1.78	4.15 ± 2.27	0.091
Cereal	n = 427	n = 63	
Low-AS Product	4.85 ± 1.84	4.21 ± 2.04	0.010
High-AS Product	3.43 ± 1.98	4.29 ± 1.98	0.002
Fruit Juice	n = 424	n = 66	
Low-AS Product	4.47 ± 1.97	3.76 ± 2.23	0.008
High-AS Product	2.34 ± 1.60	3.30 ± 2.12	0.000
Snack bar	n = 417	n = 73	
Low-AS Product	3.04 ± 1.71	2.96 ± 1.98	0.729
High-AS Product	2.37 ± 1.59	3.25 ± 2.05	0.000
Ice Cream	n = 367	n = 123	
Low-AS Product	4.35 ± 1.96	3.55 ± 2.03	0.000
High-AS Product	3.36 ± 1.88	3.96 ± 2.01	0.003

Note: Likelihood of purchase was reported on a 7-point scale (1 = very unlikely to purchase to 7 = very likely to purchase).

## Discussion

The new added sugars information is included in the updated NF label to nudge consumers toward reducing their added sugars consumption and ultimately, improve health outcomes. Acquiring the information, however, is likely a necessary condition for influencing selection and consumption decisions. While classical economic theory assumes that consumers are better off when they acquire free information as it helps them to make better decisions, a growing literature suggests there may be an incentive to avoid such information [17, 18]. To the best of our knowledge, this was the first study to experimentally test whether and under what conditions consumers wanted to access or avoid the added sugars information on the NF label.

The majority of consumers preferred to access the added sugars information rather than avoid it, which was consistent with the high levels of support for the disclosure of added sugars information observed by Khandpur, Rimm, and Moran [13]. However, a subset of consumers may actively avoid the new added sugars information, particularly for less healthy products like ice cream. The rates of added sugars information avoidance in this study were much lower than the rates of calorie information avoidance (58% preferred to avoid) found by Thunström et al. [29]. One possible explanation might be the difference in setting. In the Thunström et al. study [29], participants were asked about whether they would like calorie information for a restaurant meal. Food away from home may be viewed as more hedonic–a ‘treat’ or indulgence–in nature; in such cases, consumers may want to prioritize personal enjoyment of the food and eating experience over nutritional considerations. Conversely, nutrition may be a higher priority when purchasing foods for at-home consumption.

Contrary to our hypothesis, there was no significant difference in consumers’ decisions to access or avoid the added sugars information based on the information treatment received. One potential explanation could be that the added sugars information is relatively new to most consumers. This novelty may contribute to the high rates of accessing the added sugars information across both treatments. As discussed above, it is also possible that rates of accessing the information were higher because this study focused on products that are typically consumed at home instead of away from home. In general, consumers may be more motivated to access the nutrition facts for at-home purchases relative to away-from-home purchases, especially if they are the primary shoppers for their households and responsible for feeding others in the home. Lastly, the term added sugars on its own may have a negative connotation such that consumers were interested in learning more, even without fully understanding what added sugars are or diseases associated with their overconsumption. In this case, the provision of this additional information may have little impact on one’s decision to access/avoid the added sugars content.

The primary reason consumers reported for wanting to access the added sugars information was that it would matter to their food choices, which is consistent with results from a previous study that investigated consumer preferences for calorie labeling [26]. This finding suggests the added sugars information could help adjust food selection and/or consumption. Very few respondents indicated that they would enjoy the product more if they knew the added sugars content, implying that the added sugars information may function as an `emotional tax’ (in that the information evokes negative emotions) for some consumers [26].

Reasons for avoiding the added sugars information were more varied. Some consumers indicated the information would not matter for their food choice or that they didn’t want to think about added sugars when purchasing a particular product, while others stated knowing the information would make them feel guilty or enjoy the product less. Collectively, these results suggested that for a subset of consumers, the added sugars information had the potential to reduce the utility of their consumption experience. We also observed a subset of respondents who avoided the information because they reported knowing the added sugars content already. While we do not assess whether respondents’ knowledge is accurate, the new information may not really be “new” for some consumers.

We found that older consumers were more likely to avoid the added sugars information, which was consistent with the results of Thunström et al. [29]. A more surprising result was that respondents who had participated in a nutrition assistance program such as SNAP or WIC were more likely to avoid the added sugars information, particularly for the fruit juice and ice cream product categories. One possible explanation could be low health literacy of the NF label among SNAP-eligible respondents. Speirs et al. found that only 37% of SNAP-eligible adults had adequate health literacy, which was assessed based on their ability to answer questions using the NF label [30]. A limited understanding of the NF label may result in limited interest for the newly included added sugars information. It is also possible that SNAP recipients purchase less food from restaurants [31], so the setting where they make hedonic purchases (e.g., grocery store) may look different from households with more financial resources.

Our results also suggested that accessing the added sugars information is associated with healthier food choices. We found that information avoiders were more likely to purchase unhealthy products (in terms of added sugars levels included) than the information accessors. Our findings were consistent with Thunström et al. [29], who found that participants who avoided calorie information exhibited higher calorie intake, on average. We acknowledge that we cannot infer causality in this case as participants who chose to access the information may have been more likely to select healthier products regardless of information.

While this study makes many contributions to the literature, there are some limitations to acknowledge. First, the use of an online survey limited our ability to observe actual purchasing behavior, so there was some potential for hypothetical bias in our likelihood of purchase ratings. Future research should focus on how the inclusion of this information on the NF label influences non-hypothetical food purchases. Second, while we explored information avoidance for several product categories, more research is needed to determine if our findings generalize to other product categories, including the presence or absence of heterogeneity in avoidance behavior. Future research should also investigate the potential impact of variation of the levels of added and total sugars within brands and with similar flavors on consumer behaviors. Third, it should be noted that information access or avoidance decisions may also be influenced by brand. In this study, we held brand constant across the low and high added sugars products in each category; however, it is possible that some brands exhibit “health halos” that could impact consumers’ decision to access or avoid added sugars information. Lastly, while this study isolated the impact of the added sugars information, it should be noted that other information on the NF label like fat or protein and other product attributes such as price may influence consumers’ purchase intentions. Future research should explore how purchase intentions or actual purchasing behaviors changes when consumers have the full NF label to consider in addition to the added sugars information.

The inclusion of added sugars information was one of the major changes in the updated NF label. We found that most consumers were interested in acquiring this information, and they exhibited healthier purchasing behaviors for all product categories than information avoiders. Therefore, from a health policy and promotion standpoint, it is imperative to communicate to consumers that the added sugars information is now available on food products and emphasize the importance of the information for making healthier choices. FDA acknowledged the need for consumer education when it published the final regulations to update the NF label, particularly for the new added sugars information [32]. Educational efforts will also need to address the primary reasons individuals choose to avoid or access information to tailor messages that will resonate with consumers. Special attention and consideration should be given to more hedonic products, like ice cream, where individuals indicated a stronger preference for information avoidance. For these types of products, nutrition educators and dietary interventions may require additional emphasis on portion control strategies to promote healthful eating behaviors.

## Supporting information

S1 Table List of products with total and added sugars in grams. (DOCX)

S2 Table Primary reason for wanting added sugars content by product category (both information treatments combined). (DOCX)

S3 Table Primary reason for not wanting added sugars content by product category (both information treatments combined). (DOCX)

S1 Data (XLSX)
