# Supplementary material for: Consumers’ decisions to access or avoid added sugars information on the updated Nutrition Facts label
Source: PLoS One. 2021 Mar 29;16(3):e0249355. doi: 10.1371/journal.pone.0249355 (PMC8007016; doi:10.1371/journal.pone.0249355)
Supplement: S1 Table — (DOCX) [file pone.0249355.s001.docx]

S1 Table. List of products with total and added sugars in grams

| Category | Added sugars | Brand | Product | Total  sugars (g) | Added  sugars (g) |
| --- | --- | --- | --- | --- | --- |
| Ice Cream | High | Breyers | Mint chocolate chip | 22 | 17 |
|  | Low |  | Salted caramel swirl (no sugar added) | 5 | 0 |
| Fruit Juice | High | Ocean Spray | Cranberry juice cocktail | 25 | 23 |
|  | Low |  | 100% cranberry | 23 | 0 |
| Yogurt | High | Chobani | Strawberry | 13 | 9 |
|  | Low |  | Strawberry (less sugar) | 9 | 5 |
| Snack bar | High | Clif Bar | Blueberry crisp | 22 | 20 |
|  | Low |  | Tart cherry berry | 11 | 8 |
| Cereal | High | Post | Honey Nut Cheerios | 12 | 12 |
|  | Low |  | Original Cheerios | 2 | 2 |
