# Supplementary material for: Consumers’ decisions to access or avoid added sugars information on the updated Nutrition Facts label
Source: PLoS One. 2021 Mar 29;16(3):e0249355. doi: 10.1371/journal.pone.0249355 (PMC8007016; doi:10.1371/journal.pone.0249355)
Supplement: S2 Table — (DOCX) [file pone.0249355.s002.docx]

| **S2 Table.** Primary reason for wanting added sugars content by product category (both information treatments combined) | | | | | |
| --- | --- | --- | --- | --- | --- |
| Reason for wanting information | Yogurt  (n=418) | Cereal  (n=427) | Fruit Juice  (n=424) | Snack Bar  (n=417) | Ice Cream  (n=367) |
| The added sugars content would matter to my food   choice | 59.8% | 64.6% | 65.8% | 63.1% | 47.1% |
| I would enjoy this product more if I knew the added  sugars content | 7.4% | 8.0% | 9.0% | 7.9% | 10.4% |
| The added sugars information would not affect my  food choice, but I would be curious to know | 28.7% | 24.4% | 21.9% | 24.0% | 37.9% |
| I do not know | 1.2% | 0.9% | 0.2% | 1.9% | 1.6% |
| Other | 2.9% | 2.1% | 3.1% | 3.1% | 3% |
| Note: Reasons were not significantly different by information treatment. | | | | | |
