# Supplementary material for: Consumers’ decisions to access or avoid added sugars information on the updated Nutrition Facts label
Source: PLoS One. 2021 Mar 29;16(3):e0249355. doi: 10.1371/journal.pone.0249355 (PMC8007016; doi:10.1371/journal.pone.0249355)
Supplement: S3 Table — (DOCX) [file pone.0249355.s003.docx]

| **S3 Table.** Primary reason for not wanting added sugars content by product category (both information treatments combined) | | | | | |
| --- | --- | --- | --- | --- | --- |
| Reason for not wanting information | Yogurt  (n=72) | Cereal  (n=63) | Fruit Juice  (n=66) | Snack Bar  (n=73) | Ice Cream  (n=123) |
| I don’t want to think about added sugars when   purchasing this product | 12.5% | 19.1% | 12.1% | 16.4% | 22.8% |
| I would not want to know the added sugars content  because it would not matter to my food choice anyway | 29.2% | 23.8% | 18.2% | 28.8% | 29.3% |
| Knowing the added sugars content would make me feel   guilty about choosing a product with high added   sugars | 6.9% | 3.2% | 12.1% | 9.6% | 10.6% |
| I would enjoy this product less if I knew the added   sugars content | 13.9% | 6.4% | 7.6% | 9.6% | 11.4% |
| I knew the added sugars content of this product without   having to look at the added sugars information | 16.7% | 27.0% | 24.2% | 15.1% | 17.1% |
| Added sugars information doesn’t mean anything to me  since I do not know how much added sugars I’m   supposed to eat anyway | 5.6% | 6.4% | 7.6% | 4.1% | 2.4% |
| I do not know | 1.4% | 3.2% | 7.6% | 4.1% | - |
| Other | 13.9% | 11.1% | 10.6% | 12.3% | 6.5% |
| Notes: Responses among ‘other’ who stated that they already know the product contains a lot of added sugars were recoded as the option ‘I knew the added sugars content of this product without having to look at the added sugars information.’ The most frequent response for the option ‘other’ was that they do not purchase the product or they do not eat the product. Reasons were not significantly different by information treatment. | | | | | |
